# Flight tone characterisation of the South American malaria vector *Anopheles darlingi* (Diptera: Culicidae)

**DOI:** 10.1590/0074-02760200497

**Published:** 2021-03-12

**Authors:** Jose Pablo Montoya, Hoover Pantoja-Sánchez, Sebastian Gomez, Frank William Avila, Catalina Alfonso-Parra

**Affiliations:** 1 Universidad CES, Instituto Colombiano de Medicina Tropical, Sabaneta, Antioquia, Colombia; 2 Universidad de Antioquia, Departamento de Ingeniería Electrónica, Medellín, Antioquia, Colombia; 3 Universidad de Antioquia, Programa de Estudio y Control de Enfermedades Tropicales, Medellín, Antioquia, Colombia; 4 Universidad de Antioquia, Max Planck Tandem Group in Mosquito Reproductive Biology, Medellín, Antioquia, Colombia

**Keywords:** flight acoustics, reproduction, wing beat, flight-tone, malaria

## Abstract

**BACKGROUND:**

Flight tones play important roles in mosquito reproduction. Several mosquito species utilise flight tones for mate localisation and attraction. Typically, the female wingbeat frequency (WBF) is lower than males, and stereotypic acoustic behaviors are instrumental for successful copulation. Mosquito WBFs are usually an important species characteristic, with female flight tones used as male attractants in surveillance traps for species identification. *Anopheles darlingi* is an important Latin American malaria vector, but we know little about its mating behaviors.

**OBJECTIVES:**

We characterised *An. darlingi* WBFs and examined male acoustic responses to immobilised females.

**METHODS:**

Tethered and free flying male and female *An. darlingi* were recorded individually to determine their WBF distributions. Male-female acoustic interactions were analysed using tethered females and free flying males.

**FINDINGS:**

Contrary to most mosquito species, *An. darlingi* females are smaller than males. However, the male’s WBF is ~1.5 times higher than the females, a common ratio in species with larger females. When in proximity to a female, males displayed rapid frequency modulations that decreased upon genitalia engagement. Tethered females also modulated their frequency upon male approach, being distinct if the interaction ended in copulation or only contact.

**MAIN CONCLUSIONS:**

This is the first report of *An. darlingi* flight acoustics, showing that its precopulatory acoustics are similar to other mosquitoes despite the uncommon male:female size ratio, suggesting that WBF ratios are common communication strategies rather than a physical constraint imposed by size.

Malaria is one of the most important vector-borne diseases worldwide, with 219 million cases reported in 2017.^1^ In South America, *Anopheles darlingi* is a major malaria vector,^2^ ^,^ ^3^ being the primary neotropical malaria vector in the Amazon region of Brazil, Colombia, Peru and Venezuela.^4^ *An. darlingi* is an efficient malaria vector, able to maintain high levels of transmission even when present at low densities.^3^ Further, genetic differentiation among *An. darlingi* populations is suggested to enable adaptation of this species to a range of habitats.^4^ ^,^ ^5^ However, little is known about the basic biology of this species, and its reproductive behaviors have not been reported to date, making the control of this vector challenging.

Disruption of pre-mating reproductive behaviors has been proposed as a means to control mosquito populations by exploiting important mating-specific cues to prevent male-female interaction.^6^ ^,^ ^7^ One area of focus is precopulatory behavioral interactions between males and females. Prior to copulation, male and female mosquitoes must locate each other and interact. Males are attracted to tones produced by the female wing beat,^8^ ^,^ ^9^ although additional cues are likely to aid male-female attraction.^10^ The mating encounter site for some species is around the host (e.g., *Aedes aegypti*) - males intercept females as they attempt to blood-feed.^11^ ^,^ ^12^ Males of some anopheline species form swarms - females penetrate the swarm to find a mate;^13^ it is unknown if *An. darlingi* males form swarms.

Male and female *Ae. aegypti*, *Anopheles gambiae*, *Anopheles albimanus* and *Culex quinquefasciatus* interact acoustically pre-copula.^14^ ^,^ ^15^ ^,^ ^16^ ^,^ ^17^ ^,^ ^18^ One such interaction is rapid frequency modulation (RFM), the rapid increase of the male wing beat frequency (WBF), followed by tone oscillation, terminating with a decrease in tone frequency.^19^ ^,^ ^20^ This phenomenon occurs when males approach females during a mating attempt^19^ ^,^ ^20^ and appears to be a common male acoustic behavior during courtship, having been described in *Ae. aegypti*, *Cx. quinquefasciatus*, *An. gambiae*, *An. coluzzi*, and *An. albimanus*.^17^ ^,^ ^19^ ^,^ ^20^ ^,^ ^21^ Further, in *Ae. aegypti*, *Cx. quinquefasciatus*, and *An. gambiae* males and females modulate their WBFs to match in a shared harmonic during courtship, a phenomenon known as harmonic convergence.^14^ ^,^ ^16^ ^,^ ^18^ ^,^ ^22^

Mosquito WBFs can be an important characteristic of a species,^23^ ^,^ ^24^ ^,^ ^25^ as it mediates mating, although closely related species can have similar flight tones.^10^ In addition, WBFs are influenced by factors such as temperature, humidity and age.^22^ ^,^ ^26^ Intraspecific body size has also been suggested to influence WBF,^22^ ^,^ ^27^ although other reports find no such effect.^17^ ^,^ ^28^ Thus, how body size influences flight tones of individuals remains unclear. In addition, males and females of most species usually exhibit different wing-beat frequencies, with males producing higher frequencies. It has long been assumed that this frequency difference is associated with the smaller size of males.^9^ ^,^ ^12^ ^,^ ^29^ However, in *An. darlingi*, males and females collected in the field are similarly sized.^30^ This species therefore presents an ideal opportunity to evaluate how male-female size ratio influences mosquito acoustic interactions. Furthermore, evaluation of the acoustic mating behaviors of *An. darlingi*, an understudied species, will contribute to our knowledge of mosquito mating behaviors.

To examine precopulatory behavioral interactions in an understudied malaria vector, we characterised *An. darlingi* WBF and examined acoustic behaviors upon exposure to the opposite sex. We find that lab reared males are larger than females, unlike other mosquito species. Although larger, males broadcast a significantly higher WBF than females, showing that the WBF ratio between males and females is not determined by body size, contrary of what has been assumed in the past.^9^ ^,^ ^12^ ^,^ ^29^ Upon exposure to tethered females, males displayed RFM upon female approach. This behavior is observed in other mosquito species, showing that the communication dynamic during mating is preserved in *An. darlingi* despite the particular size of males and females of this species. These findings support the idea that the acoustic dynamic of mosquito mating behavior is a process of communication rather than a byproduct of motion. Moreover, as flight tones are currently being used in the development of novel surveillance strategies,^6^ ^,^ ^23^ ^,^ ^25^ ^,^ ^31^ ^-^ ^33^ insight into *An. darlingi* reproductive behaviors and WBF characterisation will aid their surveillance and control.

## MATERIALS AND METHODS

*Mosquitoes* - *An. darlingi* from Universidad Peruana Cayetano Heredia - ICEMR insectary (Iquitos, Peru) were used in our experiments. This colony has been maintained since 2012.^34^ ^,^ ^35^ Pupae were individualised in 5 mL tubes to ensure virginity and adults were separated by sex and transferred to sex-specific cages upon eclosion. Mosquitoes had access to 15% honey-water solution *ad libitum*. All recordings were conducted at 26**º**C and 80% relative humidity (RH). Five to seven-day-old mosquitoes were used in all experiments.

*Audio recording set up* - Mosquitoes were recorded in a 4L plastic cage using a particle velocity microphone (NR-23158-000, Knowles; Itasca, IL, USA). A USB audio interface (M-Track Quad Four Channel Audio; M-Audio, Cumberland, USA) was used to amplify and digitise recordings at a sample rate of 11025 Hz/24 bits.

*Experimental procedures* - We first recorded mosquitoes individually to determine WBFs when in free flight and when tethered. As similar aged individuals were difficult to obtain from this colony (i.e., we could not synchronise hatch rates and experienced high mortality subsequent to individualisation), the same mosquitoes were utilised to determine WBF under both conditions. Tethered mosquitoes were immobilised as in Pantoja-Sánchez et al.^17^ and placed 1 cm above the microphone to record their WBFs. Free flying mosquitoes were recorded using a rod with an adhered microphone; a researcher manually followed their flight, maintaining a distance of 5-10 cm. We next recorded free flying males and tethered females to examine acoustic interactions during a mating interaction - an immobilised female was placed ~5 cm from the microphone to allow movement around her [Supplementary data (Figure)]. Ten-fifteen males were then introduced into the cage. Females were replaced upon copulation; spermathecae were subsequently dissected to determine insemination status. We recorded all trials with a camera (FLIR-FLEA 3 1.3 MPColor USB 3 Vision with a Fujinon - FF125HA-1B 12.5 mm lens) and identified and timed behaviors in real time. Mosquito wings were measured as in van den Heuvel et al.^36^ to estimate body size.

*Signal and statistical analysis* - Flight-tone audio recordings were analysed using spectrograms (Fast Fourier transform-based, length of 4096 points, hamming window of 80 ms and 50% overlapping). To evaluate acoustic interactions, audio segments occurring during observed mating attempts were analysed. From the spectrograms, we used the male second harmonic and the female third to examine flight-tones during male-female behavioral interactions due to frequency resolution but present our results in terms of their fundamental WBFs for simplicity, as done previously.^17^ We report the time of the interaction as the length of male’s flight tone. Male and female responses were assessed by evaluating the extent of frequency modulation in the second and third harmonic, respectively. Male measurements were divided by two (ΔF = (F_max_-F_min_)/2) and female measurements by tree (ΔF = (F_max_-F_min_)/3) to express results in terms of fundamental frequency equivalent to the WBF. We further assessed female behavior by examining the rate of increase (ΔF/Δt) after male detection.

To compare frequencies between flight conditions (tethered and free flight), and to discriminate differences between sexes, we used t-tests. Normality for the variable WBF was determined with a Shapiro-Wilk test. A linear regression was used to determine the relationship between wing size and WBF. Residuals were tested for normality, homogeneity of variance and independence using Shapiro-Wilks, Bartlett and Durbin-Watson tests, respectively. We compared male-female contact and copulation interactions using the non-parametric Mann-Whitney U test to assess similarities and differences in the distributions of the variables analysed. Results are reported as mean ± standard deviation for variables that followed a normal distribution, and as median (interquartile range - IQR) for variables that did not. Signal processing was performed using Matlab (R2016a, Mathworks Inc., Natick, USA). Statistical analysis was performed using the car package^37^ of R (Vienna, Austria).^38^

## RESULTS

**Fig. 1:**
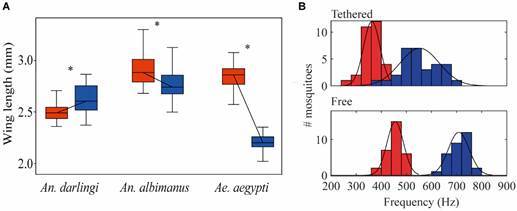
wing beat frequencies and sizes of male and female *Anopheles darlingi*. We used wing length as a proxy for body size. (A) Distribution of female (red; n = 30) and male size (blue; n =3 0). Sizes for male and female *Aedes aegypti* ^39^ and *An. albimanus* ^17^ are shown for comparison. (B) Top panel: frequency distribution of tethered females (red; n = 30) and males (blue; n = 30). Bottom panel: frequency distribution of free-flying females (red; n = 30) and males (blue: n = 30).

*Body sizes of An. darlingi adults* - Using dry weight to examine body size in field collected *An. darlingi*, Lounibos et al.^30^ reported that males and females of this species were similarly sized. As *An. darlingi* dry weight is highly correlated with wing length,^30^ we measured wing lengths to determine male and female size of our lab reared specimens. Female *An. darlingi* (2518 ± 88 μm) were significantly smaller than males (2635 ± 14 μm) (t-test: t_(29)_ = 3.82, p < 0.01; Fig. 1A) when reared under standard laboratory conditions unlike what has been observed in many species across different genera of Culicidae.^9^ ^,^ ^12^ The male/female size ratio in *An. darlingi* (size ratio 1.046) is distinct compared to other species in the same genus such as *An. albimanus* (size ratio: 0.95)^17^ or in a different genus such as *Ae. aegypti* (size ratio: 0.81)^39^ (Fig 1A).

*Wing beat frequencies of male and female An. Darling* - We recorded individual adults to characterise their flight tones under two conditions: when tethered and in free flight as some reports suggest that tethering impacts WBFs while others report no affect.^17^ ^,^ ^19^ ^,^ ^28^ ^,^ ^40^ We found significant differences between the two conditions: the mean WBF of tethered males was 553 **±** 78 Hz (n = 30), and 709 ± 42 Hz (n = 30) in free flight (t-test: t_(29)_ = -9.36, p < 0.01). The mean WBF of tethered females was 362 ± 33 Hz (n = 30), and 456 ± 33 Hz (n = 30) in free flight (t-test: t_(29)_ = -10.97, p < 0.01; Fig. 1B). Although males are larger than females (Fig. 1A), males broadcast significantly higher frequencies (1.53 times higher) than females when tethered (t-test: t_(29)_ = 12.31, p < 0.01) and 1.55 times higher in free flight (t-test: t_(29)_ = 26.10, p < 0.01) similar to what has been observed in other species such as *Ae. aegypti* and *An. albimanus*.^17^ ^,^ ^40^ Within each sex, we did not detect an effect of size on the WBF when tethered (Linear regression: males: R^2^ = 0.071, F_28_ = 2.14, t = -1.462, p = 0.1549; females: R^2^ = 0.001, F_28_ = 0.055, t = 0.23427, p = 0.8165).

*Male-female acoustic interactions during a mating attempt* - To investigate acoustic interactions between sexes, we exposed free flying males to a tethered-flying female. We analysed all interactions where the male’s flight-tone was detected. Two types of interactions were observed: (1) male-female leg contact and (2) copulation, defined as visible genitalia engagement. However, sperm transfer was never detected upon female dissection, possibly due to the inability to achieve a proper angle for insemination.^41^

**Fig. 2:**
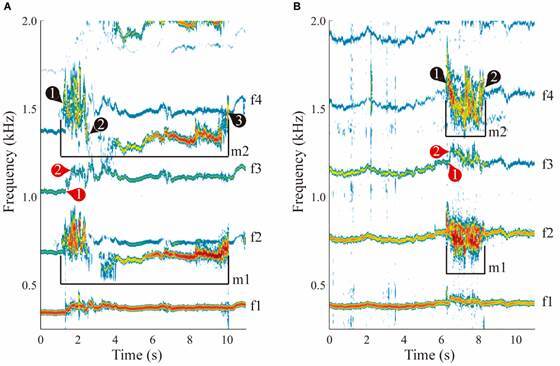
male-female spectrograms in interactions that end in copulation (A) or only contact (B). Female (f) and male (m) fundamental frequency and its harmonics are shown. Colours indicate the power of each frequency component; red is the most powerful, blue the least powerful, and white indicates the noise floor. Notable events are indicated for males (black numbers) and females (red); male signal is specified with a bracket in (A) and (B). (A) Male rapid frequency modulation (RFM) begins (black 1) and ends (black 2). In this interaction, male flight tone was stable until disengagement from the female (black 3). (B) RFM begins (black 1) and ends (black 2), with male departure shortly afterwards. In both outcomes, the female increased her wing beat frequency (WBF) upon male detection (red 1), quickly reaching peak frequency (red 2).

**Fig. 3:**
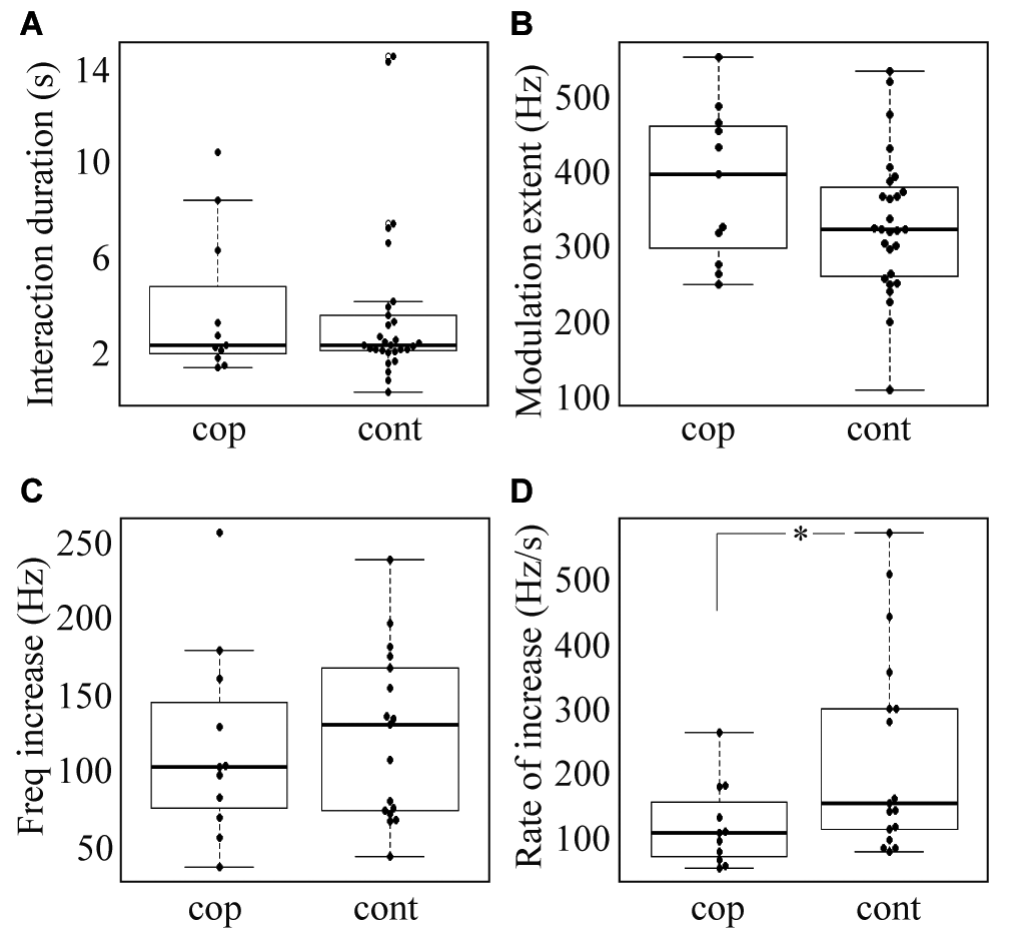
comparison between copulation (cop) and contact (cont) interaction duration (A), modulation range of male flight tone (B), frequency increase of female flight tone (C), and rate of frequency increase in female flight tone (D). Only the female rate of frequency increase significantly differed between interaction types, indicated by * (p < 0.05). Boxplots indicate the minimum, maximum, median, first quartile and third quartile in the data set.

Male signal was detected from 40 distinct interactions using 15 females (11 copulations, 29 contacts). In all interactions, when nearing the female, RFM of the male flight tone occurred followed by modulation of the female frequency (Fig. 2). In copulation interactions, the median time of male interaction was 2.35 s (n = 11, IQR 1.95 - 4.82 s), male flight-tones were characterised by RFMs with a modulation extent (F_max_-F_min_) of 396.51 Hz (IQR 296.84 - 460.69 Hz). RFM was followed by a decrease in the modulation (Fig. 2A) or by male wing beat cessation; in both cases while engaged with the female genitalia. In contact interactions, the median time of male interaction was 2.36 s (n = 29, IQR 2.13 - 3.78 s), male flight-tones were characterised by RFMs until departure from the female (Fig. 2B), with a modulation extent of 321.99 Hz (IQR 279.347 - 383.14 Hz). No differences in the interaction time (Mann-Whitney U-test: U_11,29_ = 129.00, Z = 0.92, p = 0.35; Fig. 3A) or modulation extent (Mann-Whitney U-test: U_11,29_ = 113.00, Z = -1.41, p = 0.16; Fig. 3B) were detected between interaction types.

Female flight-tones were also analysed. From the 40 interactions described above, 12 were excluded from our analysis as females stopped their wing beat upon male contact. Despite being tethered, female signal modulation was detected in the remaining 28 interactions (11 copulations, 17 contacts). Females reacted to the male approach by modulating their flight-tone frequency - we observed an initial WBF increase similar to descriptions in other mosquito species.^19^ ^,^ ^21^ The magnitude of the frequency increase in contact (median 130.38 Hz, IQR 73.18 - 167.72 Hz) and copulation interactions (median 101.78 Hz, IQR 75.19 - 144.87 Hz) was similar (Mann-Whitney U-test: U_11,17_ = 83, Z = 0.49, p = 0.62; Fig. 3C). However, the rate of frequency increase in contact interactions (median 158.46 Hz/s, IQR 117.86 - 304.40 Hz/s) was significantly higher compared to copulation interactions (median 112.67 Hz/s, IQR 75.99 - 159.94 Hz/s; Mann-Whitney U-test: U_11,17_ = 47, Z = 2.18, p = 0.028; Fig. 3D).

## DISCUSSION

Mosquito mating has been widely used as a target to develop novel control strategies.^42^ The effectiveness of such strategies, however, require a profound understanding of behaviors associated with mosquito reproduction,^42^ in which flight-tones play a major role.^21^ ^,^ ^43^ Increasing our knowledge on the mating behavior of various species provides new insights on different aspects of acoustic interactions that influence mating. This study investigates the mating behavior of a particular mosquito species: *An. darlingi.* We show how *An. darlingi* bioacoustics are similar to other species despite their uncommon intraspecific male/female size ratio.^9^ ^,^ ^12^ ^,^ ^29^

When exposed to a tethered female, male RFM occurred when approaching and contacting the female. Similar observations in *Cx. quinquefasciatus*, *An. gambiae*, *An. coluzzi* and *Ae. aegypti*, indicate the importance of this behavior.^19^ ^,^ ^20^ ^,^ ^21^ RFM duration was similar in all interactions but varied greatly during male-female contact in the absence of copulation. In *Ae. aegypti*, RFM cessation coincides with formation of the ventral-ventral copulation position.^21^ Similarly, *An. darlingi* RFM ends when a pair engage their genitalia; the male decreases the frequency modulation of his tone or ceases flying. This might be a general Anopheline behavior, as mating pairs fall to the ground after couple formation.^44^ ^,^ ^45^ Tethered *An. darlingi* females also modulated their flight tone. When males neared or made contact, tethered females increased their WBF at a higher rate than when the interaction resulted in copulation. This might be reflective of active female acceptance or rejection, although more experiments are necessary to test this hypothesis. Tethered *Culex* females modulate their WBF as a result of physical contact by the male.^19^ Whether WBF modulation by *An. darlingi* females initiates during the male approach or only upon physical contact will require higher resolution videos.

Contrary to what has long been assumed,^9^ ^,^ ^12^ ^,^ ^29^ this study shows that male-female WBF distributions of medically relevant mosquito species and acoustic interactions that occur in mating attempts are not constrained exclusively by the intraspecific male/female size ratio. Moreover, our findings support the hypothesis that the characteristics of the acoustic dynamic between males and females correspond to a communication strategy rather than a byproduct of motion. By describing the precopulatory acoustic behaviors and WBF distributions of *An. darlingi*, this study contributes to the overall knowledge of the flight acoustics of this vector and mosquitoes in general. We hope this study will promote future investigations on species of medical relevance for Latin America and aid their surveillance.
