# Supplementary material for: Flight tone characterisation of the South American malaria vector Anopheles darlingi (Diptera: Culicidae)
Source: Mem Inst Oswaldo Cruz. 2021 Mar 12;116:e200497. doi: 10.1590/0074-02760200497 (PMC7968435; doi:10.1590/0074-02760200497)
Supplement: Supplementary file 1 [file 1678-8060-mioc-116-e200497-s.pdf]

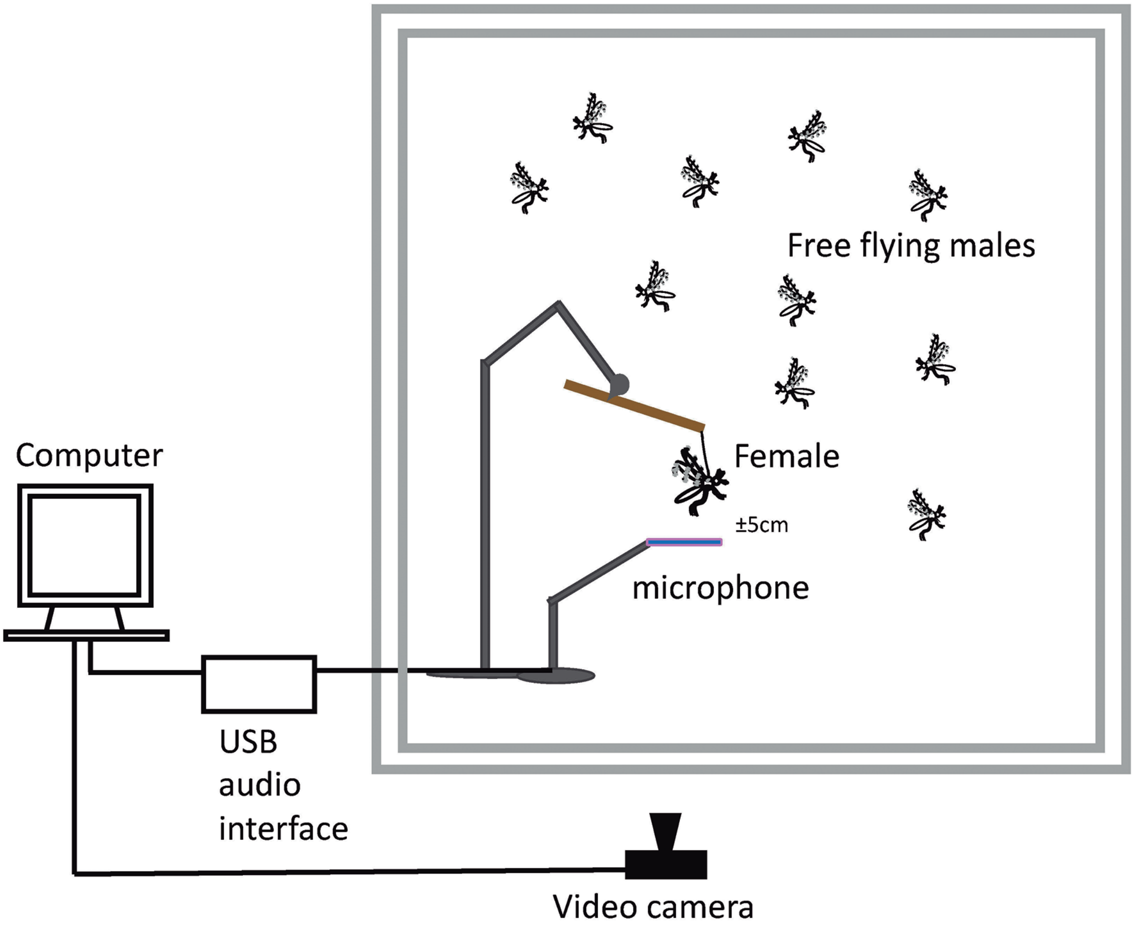

Diagram of the experimental setup for our tethered female assays. A single female is tethered with a human hair and placed above the microphone. The microphone was placed 5 cm from the female to allow for male movement around the female (10-15 males were introduced into the arena). An audio and visual record was taken for this assay.

CC BY
